# Influence of Dispersed Phase Content on the Mechanical Properties of Electroless Nanocomposite Ni-P/Si_3_N_4_ and Hybrid Ni-P/Si_3_N_4_/Graphite Layers Deposited on the AW-7075 Alloy

**DOI:** 10.3390/ma16186100

**Published:** 2023-09-06

**Authors:** Kazimierz Czapczyk, Paweł Zawadzki, Natalia Wierzbicka

**Affiliations:** 1 Faculty of Mechanical Engineering and Ship Technology, Gdansk University of Technology, G. Narutowicza 11/12, 80-233 Gdansk, Poland; 2 Faculty of Mechanical Engineering, Poznan University of Technology, Piotrowo 3, 60-965 Poznan, Poland; pawel.zawadzki@put.poznan.pl (P.Z.); natalia.wierzbicka@put.poznan.pl (N.W.)

**Keywords:** nanocomposite, silicon nitride, graphite, aluminum alloy, adhesion

## Abstract

The article presents the results of mechanical testing of Ni-P/Si_3_N_4_ nanocomposite and hybrid Ni-P/Si_3_N_4_/graphite coatings deposited on AW-7075 aluminum alloy using the chemical reduction method. In terms of mechanical testing, microhardness was measured, and surface roughness and adhesion of the coatings to the aluminum substrate were determined using the “scratch test” method. The surface morphology of the deposited layers was also analyzed using light microscopy and scanning electron microscopy. Samples made of AW-7075 aluminum alloy with electroless deposited Ni-P/Si_3_N_4_ nanocomposite, Ni-P/graphite composite and hybrid Ni-P/Si_3_N_4_/graphite coatings with different content of dispersed phases were tested, and also, for comparison purposes, the Ni-P layer that constituted the matrix of the tested materials. Reinforcing phases in the form of silicon nitride nanoparticles and graphite particles were used in the layers. The purpose of the research was a thorough characterization of the coating materials used on aluminum alloys in terms of mechanical properties. Graphite is considered in this paper as it enables the reduction of the coefficient of friction through its lubricating properties. Unfortunately, graphite is difficult to use in selected layers as the only dispersion phase, because it has much lower hardness than the Ni-P coating. For this reason, a layer with a single dispersion phase in the form of graphite will be characterized by worse mechanical properties. It is necessary to add particles or nanoparticles with hardness higher than the base Ni-P coating, e.g., Si_3_N_4_, which improve the mechanical properties of the coating. The presented analyses of the results of the conducted research complement the previous studies on selected properties of nanocomposite layers with an amorphous structure and supplement the knowledge regarding their suitability for application to aluminum machine parts.

## 1. Introduction

Aluminum alloys of various series are currently very popular in the engineering industry as materials for many structures, depending on the strength and corrosion resistance requirements. The greatest demand for aluminum alloys is noticed in shipbuilding, aviation, the automotive industry, etc. For many decades, the 5xxx series alloys have been chosen by the shipbuilding industry for the construction of offshore vessels due to their corrosion resistance and the 7xxx series for strength reasons. In the area of aerospace, 7xxx series alloys are also among the most popular construction materials. The biggest advantage of aluminum alloys, compared to steel, is the possibility of reducing the weight of the entire structure. At the moment, the use of aluminum alloys to manufacture moving machine parts is also noticeable, e.g., gear wheels made of 7075 T6 anodically oxidized alloy, e.g., in motorcycle drives. On the other hand, every moving part of a machine is subject to abrasive wear, which results in increased expectations regarding the strength and abrasion resistance of the surface layers of aluminum alloy components. Therefore, it is required to continue and extend mechanical research in order to accurately determine the suitability of new materials in the area of mechanical engineering, including an attempt to optimize their chemical compositions and microstructures depending on the expected conditions of use.

The test results prove that the surface treatment of AW-7075 aluminum alloys by depositing nickel layers using chemical reduction significantly increases the hardness of the surface layer, which translates into increased durability of the product, e.g., due to the reduction of the coefficient of friction, which was proved in the article [1]. Generally, nickel in the electroless process is deposited from an aqueous solution containing nickel salts, a reducing agent, and a substance that regulates the pH of the solution and the reaction rate. The surface of the object in such a solution is the catalyst. The deposition reaction in a bath containing NiSO_4_ and NaH_2_PO_2_ can be represented as follows:

NiSO_4_ + NaH_2_PO_2_ + H_2_O → Ni coating + NaH_2_PO_3_ + H_2_SO_4_

In addition, based on the work [2,3], it can be seen that the incorporation of particles of the dispersion ceramic phase Si_3_N_4_ into the Ni-P and Ni-B coating material causes a significant change in the morphology and degree of surface development. For the Ni-P and Ni-B layers obtained by the electroless method, the morphology is particularly characteristic due to the distinctive structure compared to the layers obtained by other methods.

**Figure 1:**
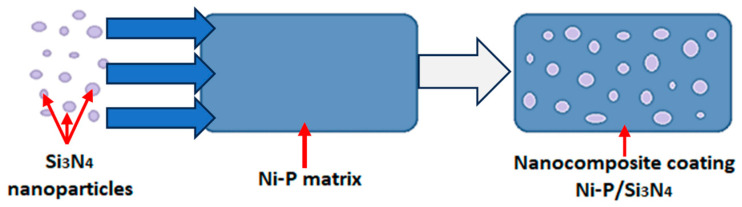
Schematic diagram of incorporation of Si_3_N_4_ nanoparticles into Ni-P matrix.

Many studies prove and indicate [4,5,6,7,8,9,10,11,12,13,14,15,16,17] that electroless nickel and boron layers can be successfully applied on a wide variety of materials and are among the best solutions to increase the resistance of the surface layer to abrasive wear and scuffing. In addition to their high hardness and good anti-wear properties, such layers also have good anti-corrosion and adhesion properties, which are particularly important in the areological system. Furthermore, to improve the properties of the coating materials, the chemical composition of Ni-P or Ni-B coatings can be modified by incorporating reinforcing phases into their structure, which can be hard or even super hard materials, i.e., materials with a hardness greater than 33 GPa, e.g., Si_3_N_4_ or SiC. The materials in the form of e.g., Si_3_N_4_ or SiC are currently used as, e.g., antiwear coatings for single and multilayer cutting blades. Apart from that they are the subjects of research in the field of material and surface engineering in order to find new applications for materials used in machine construction [18,19,20,21,22,23,24,25,26,27,28,29,30,31,32,33]. Other examples are the use of additives in the form of graphite particles or nanocomposites to increase the lubricity and durability of oils [27,28]. Silicon nitride, as a dispersed phase, is a good research material for the formation, modification and configuration of electroless composite and nanocomposite layers, especially in combination with another additive, e.g., graphite. Adhesion tests and the results of structural tests show that the Ni-P/Si_3_N_4_ nanocomposite coating can be deposited directly on steel, iron, plastic and aluminum alloys. Such layers deposited on construction materials make it possible to significantly increase the hardness and resistance of the surface to abrasive wear compared to base materials, which has been confirmed in tests, e.g., using the pin-on-disc method. Moreover, different dispersed phases can be successfully used in one coating to precisely combine and complement their individual properties, which, in turn, makes it possible to obtain a synergy effect. An example is the incorporation of hard silicon nitride particles into the layer to increase the hardness of the coating, which increases its resistance to abrasive wear [16], and graphite particles to obtain self-lubricating properties at the same time, which also contribute to reducing the coefficient of friction [7,28]. Such configuration in the form of a hybrid layer is advantageous, especially under conditions of periodically limited lubrication. The purpose of this solution is to increase the durability and reliability of manufactured parts in terms of tribology, inter alia. Unfortunately, there are some limitations that do not allow arbitrary composition of the composite coating—mainly for technological reasons at the layer deposition stage. In this study, to increase the hardness of the surface of aluminum parts, a nanocomposite Ni-P/Si_3_N_4_ (Figure 1) and a hybrid Ni-P/Si_3_N_4_/graphite coatings and, for comparison reasons, the Ni-P coating were deposited using the method of chemical reduction. Particular emphasis was placed on the two most important indicators of suitability of the tested coating materials: microhardness and adhesion of the layers to the aluminum substrate.

In composite and nanocomposite coatings, the main load is carried by the matrix. Dispersion particles oppose the movement of dislocations, which in turn causes the strengthening of the coating material. On this basis, it is assumed that the degree of matrix strengthening is proportional to the ability of the particles to oppose the movement of dislocations.

Graphite has other than hard silicon nitride (Si_3_N_4_ is super hard material) very necessary and useful properties that help reduce the wear of machine parts, which is why, in fact, graphite lubricants are often used in gears. However, in this case, graphite was added as the second minor dispersion phase mainly in order to achieve self-lubricating properties of the coating, which resulted in improving the tribological properties, directly and indirectly, the adhesion of the layer to the substrate. Another aspect that decides about undertaking this topic is the technological aspect, i.e., the difficulties associated with the incorporation of a limited amount of this type of particle (graphite or PTFE or MoS_2_) into the matrix of electroless Ni-P coatings. A novelty is an attempt to achieve a synergy effect by selecting the optimal content of Si_3_N_4_ nanoparticles (therefore, several different contents of this dispersion phase were tested) with a minimum content of graphite particles in the Ni-P coating, which is to help in obtaining such a composition of the hybrid coating composition that will be characterized by both high hardness and adhesion (due to the use of Si_3_N_4_), as well as increased resistance to abrasive wear (due to the graphite particles content) also in conditions of short-term dry friction—with insufficient presence of a lubricant. However, before the tribological tests, basic tests were performed, i.e., surface morphology and topography, microhardness and layer adhesion.

## 2. Materials and Methods

**Figure 2:**
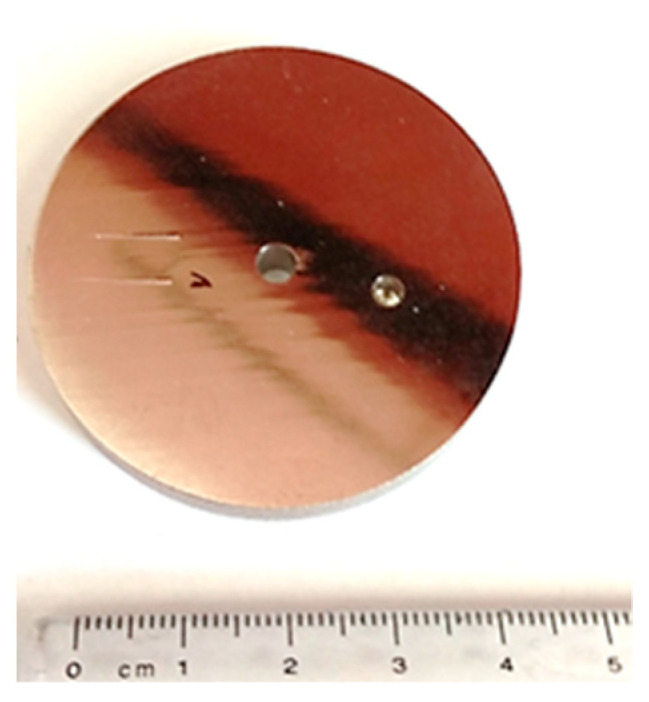
The sample of AW-7075 with electroless Ni-P/Si_3_N_4_ coating.

Laboratory tests covered the AW-7075 aluminum alloy as substrate material for the following electroless deposited coatings: Ni-P, Ni-P/Si_3_N_4_ (Figure 2), Ni-P/graphite and Ni-P/Si_3_N_4_/graphite. The chemical composition of aluminum alloy AW-7075 is presented in Table 1.

**Figure 3:**
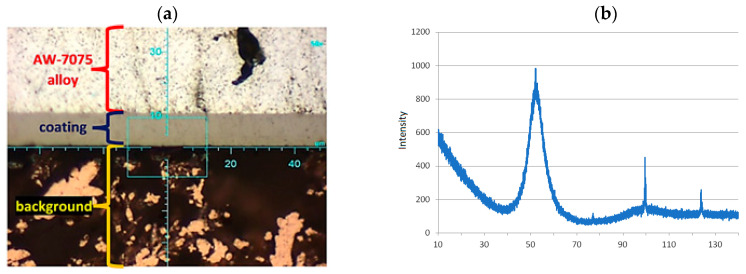
The elementary research results containing microscopic image of the Ni-P/Si_3_N_4_ (5 g/dm^3^) cross-section with lens 50× (**a**) and diffraction patterns of Ni-P alloy coating (**b**).

The technological quality of the aluminum substrate and the deposited coatings, i.e., morphology, microhardness, chemical composition and roughness, as well as the quality of the transition layer, i.e., adhesion of the layers to the substrate, were tested in the areological system under consideration. The dimensions of the AW-7075 alloy samples were as follows: diameter D = 50 mm and thickness g = 7 mm. Before the deposition of the Ni-P layers, the surfaces of the samples were degreased in an organic solvent, etched in an alkaline solution and galvanized in a multi-component solution. For the formation of Ni-P layers by chemical reduction, a multi-component bath was prepared with the following composition: NiSO_4_, reducer (NaH_2_PO_2_) and buffer (C_2_H_3_NaO_2_) to stabilize the reaction at 4.3–4.6. The details of composition and concentrations are given in Table 2 and Table 3. The bath temperature during the deposition process was 363 K. The thickness of the coatings was the same and was: 10 µm ± 2 µm, which was obtained by appropriately selected deposition time (60 min) in the electroplating baths. Also, the actual thickness of the layers was verified by microscopic examination of the cross-sections of the samples, i.e., after their cutting and preparation of metallographic specimens, as shown in Figure 3.

### 2.1. Surface Morphology and Roughness

The surface layers of the samples were characterized by qualitative surface image assessment, i.e., morphology tests were performed using the Keyence VHX 5000 optical microscope and a JEOL JSM-7800F scanning electron microscope. The surface morphology tests were carried out by SEM with the following parameters: 10 mm working distance and 15 keV. In addition, the structure of the alloy layer was analyzed by X-ray diffraction using the MiniFlex II Rigaku device. The surface roughness parameters were examined with an optical profilometer (Alicona IF-Portable RL) and the results are presented in Table 2. The tests were performed to characterize the surface layers of the deposited materials qualitatively and quantitatively. Profilometry analyses covered a square surface with dimensions of 1.014 × 1.014 mm in the central part of the sample.

### 2.2. Microhardness Testing

Microhardness tests were performed for the AW-7075 alloy, Ni-P, Ni-P/Si_3_N_4_ and Ni-P/Si_3_N_4_/graphite layers deposited on the AW-7075 alloy. The tests were done using the Vickers method using the semi-automatic FM 800 microhardness tester (indenter load: 250 [mN], load duration: 10 [s]) according to PN-EN ISO 6507-1:2018-05 [35]. The averaged measurement results are presented in Table 3. For the substrate material and the deposited layers, 4 measurements were taken for each sample.

### 2.3. Adhesion Testing

Adhesion tests of the coatings deposited on the AW-7075 alloy were performed using the scratch test method according to PN-EN ISO 20502:2016-05 [36]. The Revetest device by CSEM with a Rockwell indenter was used for the tests with an increasing progressive load from 1 to 100 [N] and a constant speed of the indenter displacement: 10 [mm/min]. The length of each scratch was 10 [mm]. Two scratches were made on each sample and the acoustic emission signal, friction force, friction coefficient and normal force were recorded simultaneously during the measurements. Detailed analyses of the course of scratch formation and adhesive and cohesive cracks were carried out using the Keyence VHX 5000 microscope.

## 3. Results

All samples made of the AW-7075 aluminum alloy had a chemical composition, according to the data in Table 1. The results of the measurements are presented according to the order of the performed tests, i.e., morphology, roughness, microhardness, and adhesion.

### 3.1. Layers Characteristics

**Figure 4:**
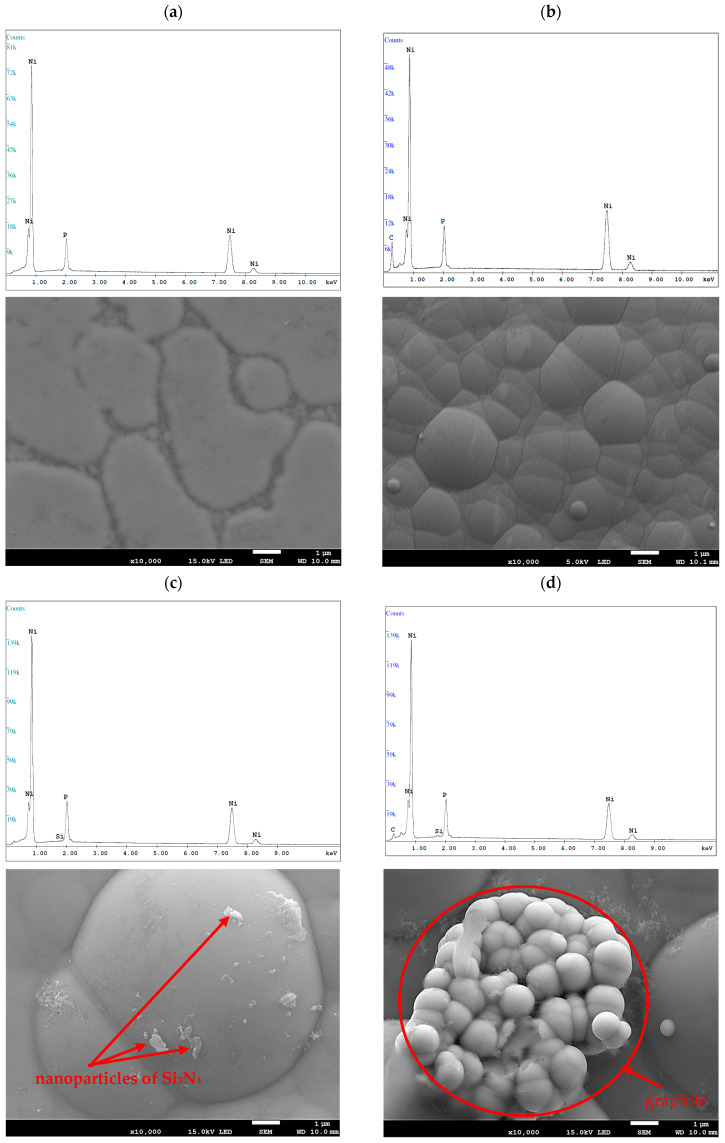
Examples of SEM images of the layers (lens: 10.000×). [(**a**)—Ni-P, (**b**)—Ni-P/graphite (0.5 g/dm^3^), (**c**)—Ni-P/Si_3_N_4_ (1 g/dm^3^), (**d**)—Ni-P/Si_3_N_4_/graphite ((1 + 0.5) g/dm^3^)].

**Figure 5:**
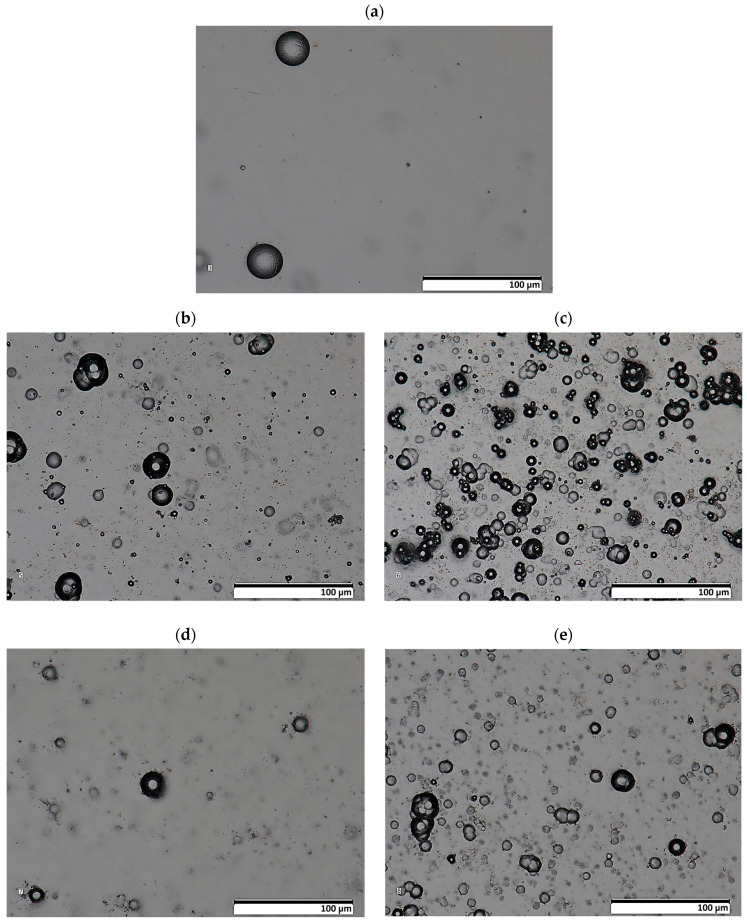
Images of the morphology of Ni-P and Ni-P/Si_3_N_4_ layers (lens: 1.000×). [(**a**)—Ni-P, (**b**)—Ni-P/Si_3_N_4_ (0.5 g/dm^3^), (**c**)—Ni-P/Si_3_N_4_ (1 g/dm^3^), (**d**)—Ni-P/Si_3_N_4_ (2 g/dm^3^), (**e**)—Ni-P/Si_3_N_4_ (5 g/dm^3^)].

**Figure 6:**
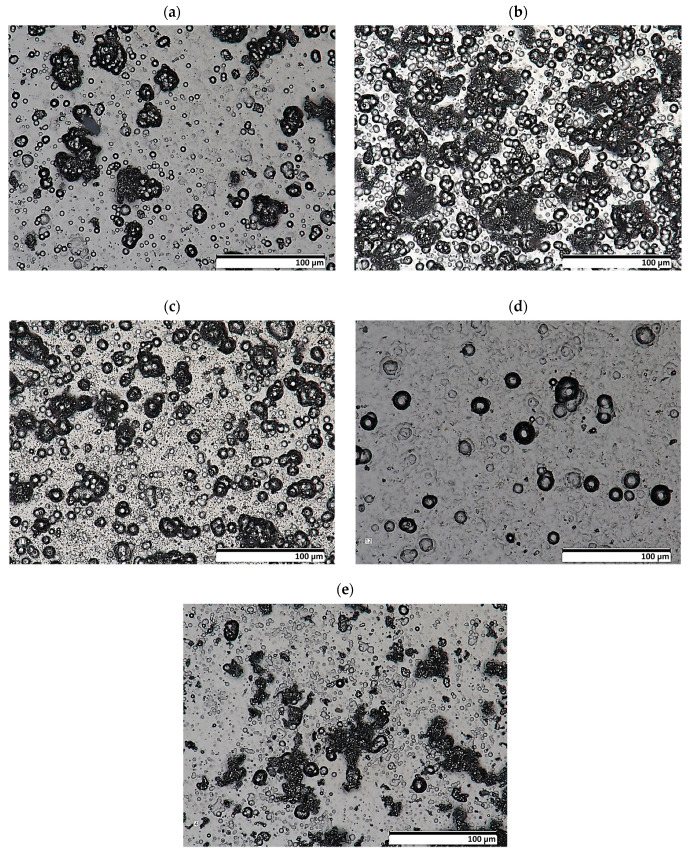
Images of the morphology of Ni-P/Si_3_N_4_/graphite and Ni-P/graphite layers (lens: 1000×). [(**a**)—Ni-P/Si_3_N_4_/graphite ((0.5 + 0.5) g/dm^3^), (**b**)—Ni-P/Si_3_N_4_/graphite ((1 + 0.5) g/dm^3^), (**c**)—Ni-P/Si_3_N_4_/graphite ((2 + 0.5) g/dm^3^), (**d**)—Ni-P/Si_3_N_4_/graphite ((5 + 0.5) g/dm^3^), (**e**)—Ni-P/graphite (0.5 g/dm^3^)].

**Figure 7:**
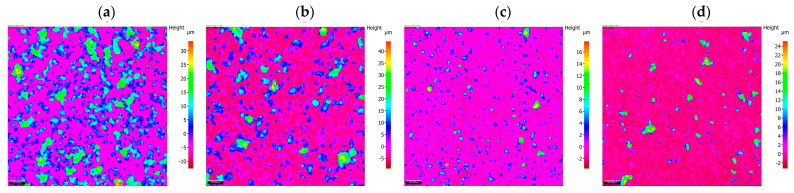
Surface topography of hybrid layers with increasing content of Si_3_N_4_ particles. [(**a**)—Ni-P/Si_3_N_4_/graphite ((0.5 + 0.5) g/dm^3^), (**b**)—Ni-P/Si_3_N_4_/graphite ((1 + 0.5) g/dm^3^), (**c**)—Ni-P/Si_3_N_4_/graphite ((2 + 0.5) g/dm^3^), (**d**)—Ni-P/Si_3_N_4_/graphite ((5 + 0.5) g/dm^3^)].

**Figure 8:**
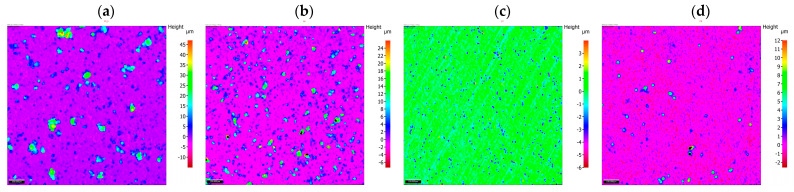
Surface topography of nanocomposite layers with increasing content of Si_3_N_4_ particles [(**a**)—Ni-P/Si_3_N_4_ (0.5 g/dm^3^), (**b**)—Ni-P/Si_3_N_4_ (1 g/dm^3^), (**c**)—Ni-P/Si_3_N_4_ (2 g/dm^3^), (**d**)—Ni-P/Si_3_N_4_ (5 g/dm^3^)].

**Figure 9:**
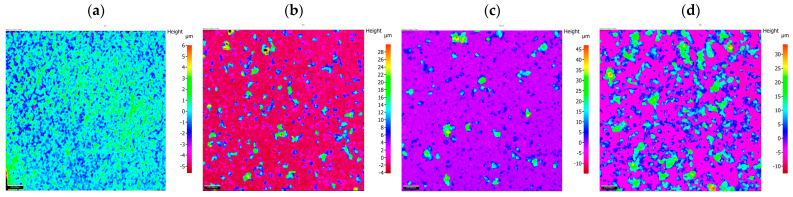
Influence of additives on the surface topography of deposited layers [(**a**)—Ni-P, (**b**)—Ni-P/graphite (0.5 g/dm^3^), (**c**)—Ni-P/Si_3_N_4_ (0.5 g/dm^3^), (**d**)—Ni-P/Si_3_N_4_/graphite ((0.5 + 0.5) g/dm^3^)].

The results of the morphology of the Ni-P, Ni-P/Si_3_N_4_, Ni-P/graphite and Ni-P/Si_3_N_4_/graphite layers with a thickness of 10 µm are presented in Figure 3, Figure 4, Figure 5 and Figure 6, while the surface topography of the layers and the roughness parameters are shown in Figure 7, Figure 8 and Figure 9. and Table 4.

The structure of the coating depends on the type of layer and its chemical composition [3]. The material of the layers formed by chemical reduction was a solid solution of phosphorus in Ni-P nickel-containing 10 wt.% P. The image showing a cross-sectional view of the sample where the coating was deposited on the aluminum substrate, together with the actual layer thickness is presented in Figure 1. The images of the surface of the layers obtained with the use of a scanning electron microscope are shown in Figure 4. The structure of the surfaces of the tested nanocomposite layers was similar, due to the dispersed phase content. The same correlation was observed for the hybrid coatings. The coatings were characterized by a compact, homogeneous structure and even deposition. The coatings on all surfaces of the samples were of a constant thickness of 10 µm. No defects were observed in the layer materials in the form of cracks, inclusions or localized delamination.

The preliminary results of the microscopic examination (Figure 4, Figure 5 and Figure 6) of the sample cross-sections confirmed the assumed thickness of the deposited coatings, which was 10 µm (Figure 3a). X-ray diffraction studies showed that the Ni-P material, on the basis of which the nanocomposite and hybrid coatings were created, has a mixed amorphous-nanocrystalline structure (Figure 3b). The largest peak indicates the amorphous-crystalline structure, while the smaller peaks in the diffraction pattern refer to the crystalline phase.

In the presented studies of profiles and surface roughness, two- and three-dimensional analysis was used. The parameters that are most often used in industrial conditions were selected, while the analyses and descriptions of the roughness results mainly included elements related to the peaks and valleys of given profiles.

Analyses of surface topography parameters were performed for samples with deposited nanocomposite and hybrid layers, as well as, for comparison purposes, for a sample with a Ni-P coating without a dispersed phase and a sample of AW-7075 aluminum alloy with no deposited layer. The thickness of all analyzed coatings was 10 μm (Table 4).

An unambiguous determination of the influence of the composition of the dispersed phase on the values of the parameters Sq and Sa is not possible. There is a noticeable tendency for the values of those parameters to increase when small amounts of the reinforcing phase components are added, i.e., 0.5 g of graphite and 0.5 g and 1 g of Si_3_N_4_ nanoparticles (Figure 7). Taking Sq = 0.889 (Ni-P) as the reference value. Adding graphite (Ni-P/graphite (0.5 g) resulted in a significant increase in this value to 4.721. In the case of samples from the Ni-P/Si_3_N_4_ group (0.5; 1; 2; 5 g), after an initial significant increase (Sq = 4.971 for 0.5 g), along with the increase in the additive content, the value of the parameter decreased to the level of Sq = 0.602 for 2 g and Sq = 0.655 for 5 g of Si_3_N_4_ nanoparticles (Figure 6). Adding graphite (0.5 g) to the Ni-P/Si_3_N_4_ layer results in an increase in the value of Sq, where, in comparison with the Ni-P/Si_3_N_4_ layer, the following was observed for all analyzed coatings—except for one coating: Ni-P/Si_3_N_4_/graphite ((5 + 0.5) g). The analysis of the parameters S10z and Sz indicates an increase in the dispersion of the values of the valleys and peaks for coatings containing 0.5 g of graphite or 0.5 g of Si_3_N_4_. Increasing the content of graphite and Si_3_N_4_ nanoparticles in the composite and hybrid coatings stabilizes the value of Sz and S10z at a level similar to that of the Ni-P coating.

In the case of the formation of coatings where a symmetric surface is required, the parameter Ssk (surface asymmetry coefficient) is very important. Significant differences are noticeable for this parameter. When analyzing the data, it was observed that the smallest asymmetry values were obtained for Ni-P/Si_3_N_4_ (2 g) (Ssk = 0.156) and Ni-P (Ssk = 1.389) samples. The Ni-P/Si_3_N_4_ (1 g) and Ni-P/Si_3_N_4_/graphite ((5 + 0.5) g) samples are characterized by the greatest asymmetry. Positive skewness was observed in all coatings, in contrast to the non-coated aluminum alloy: Ssk = −0.114. A significant increase in parameters was observed for the Ni-P/graphite content (0.5 g); although, in this case, the Ssk value decreases causing the dominance of roughness deviations in a direction greater than in the case of Ni-P coatings. After taking into account the microhardness and adhesion of coatings, lower Ssk values may indicate the potential use of selected layers in sliding connections. Adding Si_3_N_4_ (0.5 g) to the Ni-P coating results in similar surface structure properties as in the case of Ni-P/graphite (0.5 g). Increasing the amount of Si_3_N_4_ decreases the values of all parameters except for Ssk, which significantly increases. The increasing deviation of those values indicates an uneven distribution of maximum heights. For Ni-P/graphite (0.5 g), Ni-P/Si_3_N_4_/graphite ((0.5 + 0.5) g) and Ni-P/Si_3_N_4_/graphite ((0.5 + 1) g) similar parameter values were recorded. The effect of increasing the Si_3_N_4_ content in the Ni-P/Si_3_N_4_/graphite composition on the reduction of surface roughness with a clear increase in the Sku value is noticeable (Figure 7).

The last analyzed parameter was the surface inclination factor Ssq, which enables the detection of surface defects. In the case of aluminum alloy (Ssq = 2.877), the distribution of the profile ordinates is close to a normal distribution, while for the other samples, the distribution is slender, which means the presence of high peaks and deep valleys. Lower values were observed for Ni-P/graphite (0.5 g), Ni-P/Si_3_N_4_ (2 g), Ni-P/Si_3_N_4_/graphite ((0.5 + 0.5) g), Ni-P/Si_3_N_4_/graphite ((1 + 0.5) g) (up to Ssq = 10), while higher for Ni-P/Si_3_N_4_ (1 g), Ni-P/Si_3_N_4_ (5 g) and Ni-P/Si_3_N_4_/graphite ((5 + 0.5) g) (above Ssq = 146). The change in the distribution and intensity of the peaks can be observed in all graphics presenting the surface topography (Figure 7, Figure 8 and Figure 9).

Based on the measurement results, nanocomposite and hybrid coatings show similar values of roughness parameters but are highly dependent on the quantitative composition of the dispersed phase. The dominance of graphite in the chemical composition of the coating material, both in the composite and hybrid coatings, negatively affects the surface topography properties, although the exact extent to which this determines the tribological suitability cannot be determined at this stage.

In general, the results of the conducted tests indicate that the presence of Si_3_N_4_ in the dispersed phase composition, above 2 g, guarantees the best surface topography.

Surface roughness primarily affects fatigue life and contact problems [37]. Irregularities on the coating surface can lead to increased friction, especially when interacting with another rough surface. When a material is miniaturized, the effect of surface roughness influences its mechanical properties. This is because rough surfaces act as a stress concentration centers, thereby reducing the endurance limit. Surface roughness can impact the fatigue life of coatings by acting as stress concentrators. Stress concentrations tend to occur at the peaks and valleys of rough surfaces, leading to localized stress intensities and potential initiation points for cracks.

### 3.2. Layer Microhardness

The Vickers microhardness test was performed for aluminum alloy, nickel, nanocomposite and hybrid layers, the results are presented in Table 5. Ni-P, Ni-P/Si_3_N_4_ and Ni-P/Si_3_N_4_/graphite coatings showed several times higher hardness compared to the AW-7075 alloy. As the dispersed phase content of the tested coatings changed, their hardness also changed. The Vickers microhardness values for the group of nanocomposite layers and separately for the group of hybrid layers are similar; however, detailed analyses of the results indicate predominance of the Ni-P/Si_3_N_4_ (2 g) layer among the nanocomposite coatings and predominance of the Ni-P/Si_3_N_4_/graphite ((2 + 0.5) g) coating among the hybrid layers, for which the highest microhardness values were observed. Based on the results of the microscopic analyses of the areas where the indentations were made, and on the results of microhardness tests of the coatings in the cross-sections of the metallographic specimens, the tested layers were not pierced with a Vickers indenter during microhardness tests of the surfaces of the samples, also, no influence of the substrate on the final results was observed. It was found that there was a general increase in the microhardness of the surface layer with the introduction of dispersed phases, compared to nickel layers without any reinforcing phase. However, the highest values were observed for coatings deposited in a galvanic bath with a silicon nitride content of 2 g, both for nanocomposite and hybrid layers (with a clear indication of hybrid ones). Further increases in the dispersed phase content result in a minimally negative effect on the mechanical properties of the whole layer. To sum up, the Ni-P/Si_3_N_4_/graphite ((2 + 0.5) g) layer has the best mechanical properties.

**Figure 10:**
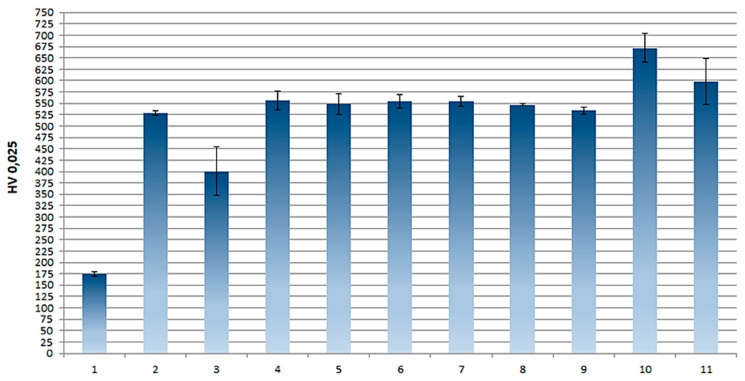
A diagram of the layers microhardness [(**1**)—7075 alloy, (**2**)—Ni-P, (**3**)—Ni-P/graphite, (**4**)—Ni-P/Si_3_N_4_ (0.5 g/dm^3^), (**5**)—Ni-P/Si_3_N_4_ (1 g/dm^3^), (**6**)—Ni-P/Si_3_N_4_ (2 g/dm^3^), (**7**—Ni-P/Si_3_N_4_ (5 g/dm^3^), (**8**)—Ni-P/Si_3_N_4_/graphite ((0.5 + 0.5) g/dm^3^), (**9**)—Ni-P/Si_3_N_4_/graphite ((1 + 0.5) g/dm^3^), (**10**)—Ni-P/Si_3_N_4_/graphite ((2 + 0.5) g/dm^3^), (**11**)—Ni-P/Si_3_N_4_/graphite ((5 + 0.5) g/dm^3^)].

The averaged measurement results are presented in Table 5. Based on this, a graphical presentation and interpretation of the obtained results were also performed by drawing up a bar chart of the microhardness of the layers under study, shown in Figure 10.

Microhardness can synthetically display the elasticity, plasticity, and strength of materials. Moreover, microhardness tests provide information about the local surface properties of a material, rather than its bulk properties. Obtained values can be correlated with the yield strength or hardness of the material, giving insights into its resistance to deformation or penetration. The microhardness of coatings can influence the coefficient of friction between the coating surface and the opposing surface. The high microhardness of a coating typically leads to a lower coefficient of friction, resulting in less energy lost due to friction. Hard coatings can exhibit greater resistance to wear, which is particularly important in applications involving high mechanical loads and intense friction. Coatings with appropriate microhardness can create a smooth and uniform surface, promoting better lubricant distribution and reducing friction [38,39,40].

### 3.3. Layer Adhesion

**Figure 11:**
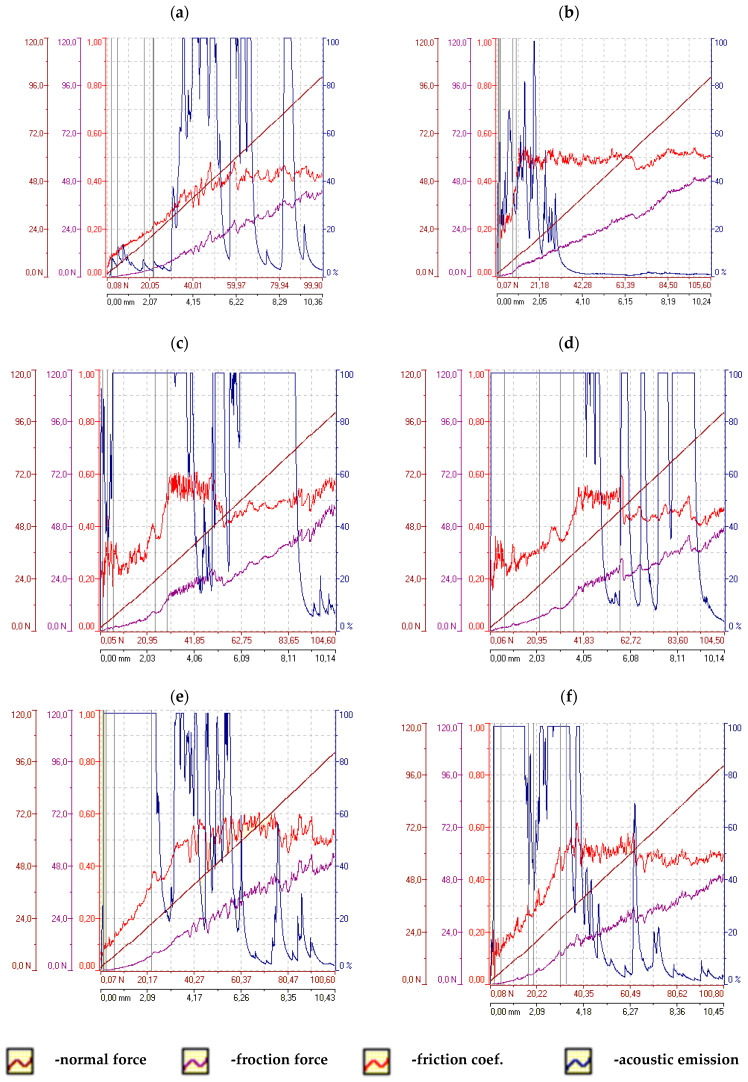
The scratch test results for samples with Ni-P and composite layers. (**a**)—Ni-P, (**b**)—Ni-P/graphite, (**c**)—Ni-P/Si_3_N_4_ (0.5 g/dm^3^), (**d**)—Ni-P/Si_3_N_4_ (1 g/dm^3^), (**e**)—Ni-P/Si_3_N_4_ (2 g/dm^3^), (**f**)—Ni-P/Si_3_N_4_ (5 g/dm^3^).

**Figure 12:**
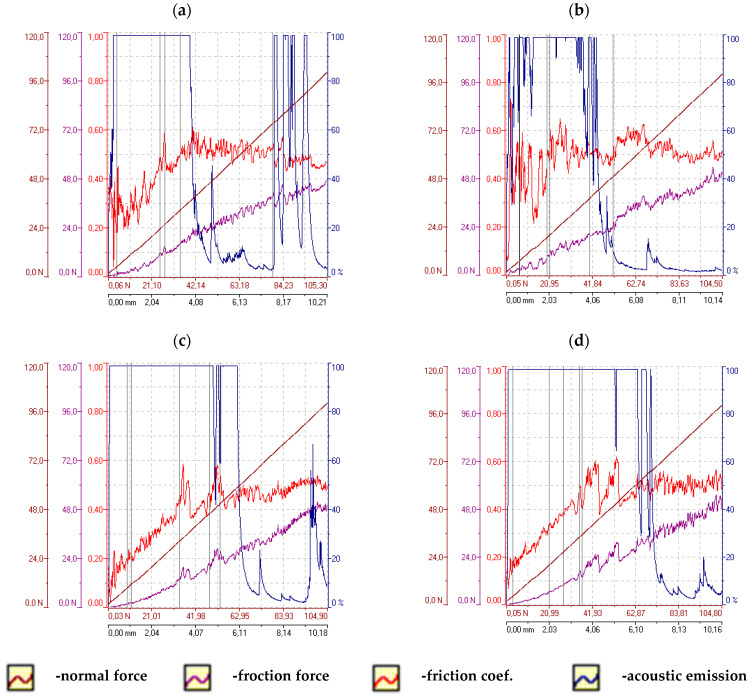
The scratch test results for samples with hybrid layers (**a**)—Ni-P/Si_3_N_4_/graphite ((0.5 + 0.5) g/dm^3^), (**b**)—Ni-P/Si_3_N_4_/graphite ((1 + 0.5) g/dm^3^), (**c**)—Ni-P/Si_3_N_4_/graphite ((2 + 0.5) g/dm^3^), (**d**)—Ni-P/Si_3_N_4_/graphite ((5 + 0.5) g/dm^3^).

**Figure 13:**
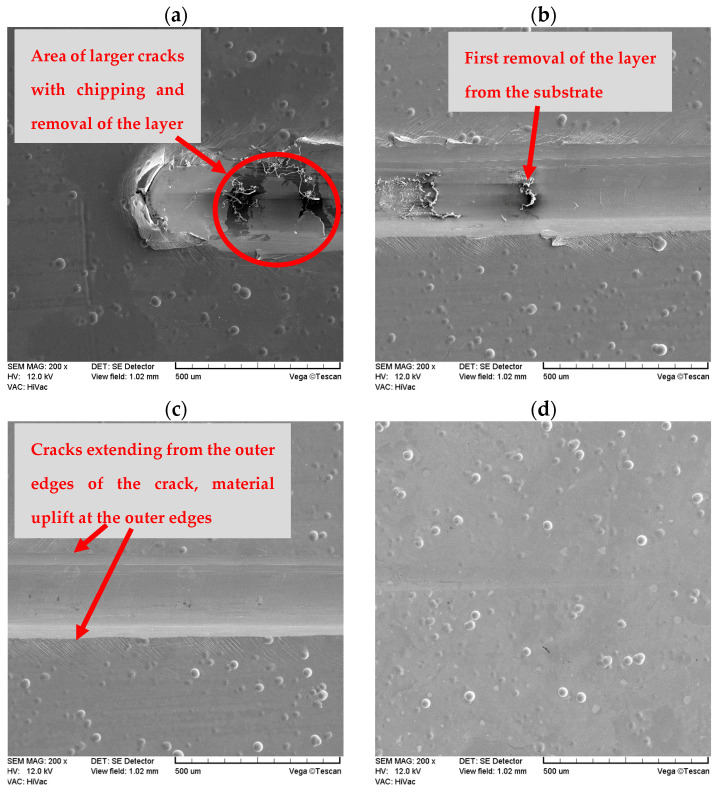
Example SEM images of the scratch in four stages on the same Ni-P layer after the test [(**a**)—the 4th (last) step of the test, (**b**)—the 3rd step of the test, (**c**)—the 2nd step of the test, (**d**)—the 1st step of the test].

**Figure 14:**
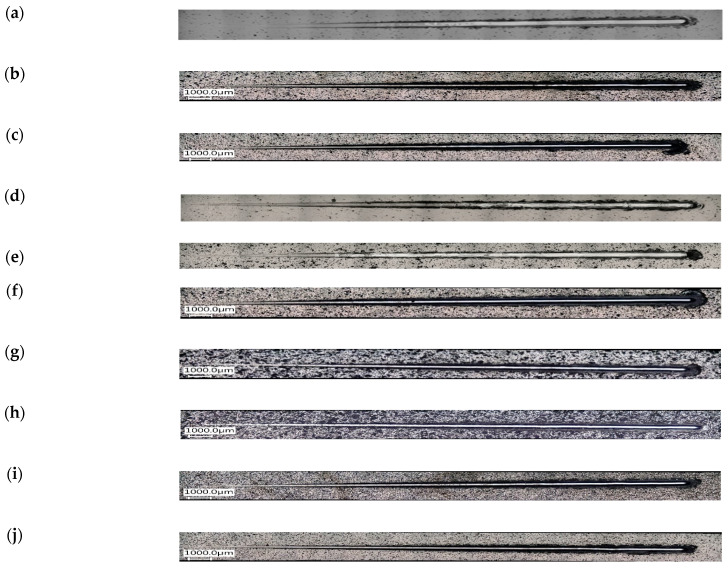
Images of the scratches on the layers after scratch tests. [(**a**)—Ni-P, (**b**)—Ni-P/Si_3_N_4_ (0.5 g/dm^3^), (**c**)—Ni-P/Si_3_N_4_ (1 g/dm^3^), (**d**)—Ni-P/Si_3_N_4_ (2 g/dm^3^), (**e**)—Ni-P/Si_3_N_4_ (5 g/dm^3^), (**f**)—Ni-P/graphite, (**g**)—Ni-P/Si_3_N_4_/graphite ((0.5 + 0.5) g/dm^3^), (**h**)—Ni-P/Si_3_N_4_/graphite ((1 + 0.5) g/dm^3^), (**i**)—Ni-P/Si_3_N_4_/graphite ((2 + 0.5) g/dm^3^), (**j**)—Ni-P/Si_3_N_4_/graphite ((5 + 0.5) g/dm^3^)].

Changes in the parameters of the scratch process, such as normal force, friction force, friction coefficient and acoustic emission along the scratch during the scratch test are presented in Figure 11 and Figure 12. An example of the sample surface with the SEM image of the area from where the layer was removed is shown in Figure 13 and general images of the scratches are shown in Figure 14. Ni-P/Si_3_N_4_ nanocomposite layers obtained in a bath with the content of the Si_3_N_4_ phase at the level of 1 and 2 g/dm^3^ were characterized by greater adhesion to the substrate compared to Ni-P and Ni-P/graphite layers, as well as Ni-P/Si_3_N_4_ layers obtained in a bath with phase content of: 0.5 and 5 g/dm^3^. Ni-P/Si_3_N_4_ nanocomposite layers with the content of the Si_3_N_4_ phase in the 5 g/dm^3^ bath showed the weakest adhesion to the substrate compared to all other tested layers. In turn, the Ni-P/Si_3_N_4_/graphite hybrid layers showed the greatest adhesion to the substrate, which was clear and noticeable based on the critical load results and when the coatings were completely removed (Table 6), as well as based on the accompanying graphs (Figure 11 and Figure 12), where the acoustic emission signal is most stable. The detailed descriptions of the scratch tests for all types of the layers are presented in Table 7, Table 8, Table 9, Table 10, Table 11, Table 12, Table 13, Table 14, Table 15 and Table 16. It was observed that the addition of graphite to the nanocomposite coating contributes to an increase in adhesion from 20 to 100%—compared to the Ni-P/Si_3_N_4_ layers. However, the mere addition of graphite, as the only dispersed phase in the Ni-P coating, negatively affects the mechanical properties and contributes to the reduction of layer adhesion by 12.5% in relation to the Ni-P coating. It was noticed that the Ni-P/graphite layer was characterized by the lowest adhesion among all tested coatings. However, by combining two different dispersed phases in the form of Si_3_N_4_ and graphite (hybrid coatings), a synergy effect was obtained in terms of all mechanical properties, which was fully noticeable based on the results of the adhesion tests. No delamination of the coatings was observed in any of the scratch tests. During each test, clear acoustic signals were recorded, which, thanks to detailed microscopic observations, made it possible to accurately determine the behavior of the material of the layers, as well as the strength and resistance to cracking. Based on the performed analyses, Table 4 presents the critical loads Lc1 and Lc2 relating to damage in the form of cohesive and adhesive cracks, respectively, for each tested layer. Additionally, the area of complete removal of the layers was determined and taken into account. To sum up, based on the conducted comparative analysis of the obtained results of adhesion tests, it was shown that the highest critical loads of Lc2 can be transferred by the Ni-P/Si_3_N_4_/graphite ((5 + 0.5) g) hybrid layer.

## 4. Conclusions

Nickel Ni-P, nanocomposite Ni-P/Si_3_N_4_ and hybrid Ni-P/Si_3_N_4_/graphite layers of an amorphous structure deposited by chemical reduction on the AW-7075 aluminum alloy are compact and are also characterized by good adhesion to the substrate material. Nanocomposite and hybrid layers show a greater degree of surface development compared to the Ni-P layer. The incorporation of dispersed phases in the form of Si_3_N_4_ powder with nanometric particle sizes, as well as graphite in the Ni-P matrix, contributes to the improvement of the mechanical properties of the layers. Nanocomposite and hybrid coatings are characterized by higher hardness than layers without a dispersed phase, i.e., Ni-P. The presence of Si_3_N_4_ nanoparticles along with graphite has a positive effect on the mechanical properties and adhesion to the substrate of the tested layers; however, the presence of graphite alone in the coating has a negative effect on adhesion, and both adhesion and microhardness are noticeably lower, even compared to the basic comparative Ni-P coating.

Furthermore, despite the fact that the most favorable results of profilometric testing were obtained for coatings with Si_3_N_4_ content of more than 2 g, it must be stressed that the profilometric tests were performed for technological quality (a priori), i.e., the state of the samples after the completion of their production. Only for the functional quality (a posteriori), i.e., the state of the product during its exploitation, i.e., after tribological tests are taken into account, it will be possible to determine the real impact of individual surface topographies on suitability in terms of operation.

In general, on the basis of this work, four elementary points referring to the test results and providing the basis for further measurements on electroless hybrid coatings containing silicon nitride and graphite were distinguished:

- Enrichment of the Ni-P electroless coating with an amorphous structure with the content of Si_3_N_4_ nanoparticles and graphite particles helps to improve the basic mechanical properties of the layer.
- The chemical reduction method used for the deposition of Ni-P/Si_3_N_4_/graphite hybrid layers allows for the incorporation of any content of Si_3_N_4_ nanoparticles and limited content of graphite particles.
- The content of two dispersion phases enables the increase of adhesion of the layer to the substrate made of AW-7075 aluminum alloy compared to the coating with one dispersion phase.
- Excessive content of silicon nitride nanoparticles results in a decrease in the microhardness value of the layers.

The results of the presented research provide a good basis for modifying the properties of components made of aluminum alloys by surface treatment consisting of applying nanocomposite and hybrid alloy layers on the components by chemical reduction, which makes it possible to increase the functionality of finished products.

## Figures and Tables

**Table 1:** Chemical composition of AW-7075 alloy [34].

Chemical Composition [%]										
Zn	Mg	Cu	Fe	Si	Mn	Cr	Zr	Ti	other	Al
5.1–6.1	2.1–2.9	1.2–2.0	max 0.50	max 0.4	max 0.3	0.18–0.28	max 0.25	max 0.20	max 0.05	the rest

**Table 2:** Components concentrations of multi-constituent substance for galvanizing.

Substrate	Chemical Formula	Concentration [g/dm^3^]
Sodium hydroxide	NaOH	120
Zinc oxide	ZnO	12
Nickel (II) sulfate	NiSO_4_ × 6H_2_O	1.5
Iron (III) chloride	FeCl_3_ × 6H_2_O	2
Sodium potassium tartrate	KNaC_4_H_4_O_6_ × 4H_2_O	15
Sodium citrate	C_6_H_5_O_7_Na_3_ × H_2_O	15

**Table 3:** Components concentrations of nickel deposition bath.

Substrate	Chemical Formula	Concentration [g/dm^3^]
Monosodium phosphate (I) (reducer)	NaH_2_PO_2_ × H_2_O	30
Sodium acetate	CH_3_COONa × 3H_2_O	35
Nickel (II) sulfate	NiSO_4_ × 6H_2_O	28
Lactic acid (pH stabilizing buffer)	C_2_H_4_OHCOOH	20

**Table 4:** Results of surface topography measurements of nickel, nanocomposite and hybrid coatings.

Parameter, µm	Ni-P (10 μm)	Ni-P/Graphite (0.5 g)	Ni-P/Si_3_N_4_ (0.5 g)	Ni-P/Si_3_N_4_ (1 g)	Ni-P/Si_3_N_4_ (2 g)	Ni-P/Si_3_N_4_ (5 g)	Ni-P/Si_3_N_4_/Graphite ((0.5 + 0.5) g)	Ni-P/Si_3_N_4_/Graphite ((1 + 0.5) g)	Ni-P/Si_3_N_4_/Graphite ((2 + 0.5) g)	Ni-P/Si_3_N_4_/Graphite ((5 + 0.5) g)	Aluminum
**Sq**	0.89	4.72	4.97	0.89	0.60	0.66	6.16	5.60	1.17	0.62	0.38
**Sz**	23.58	38.37	62.39	26.52	11.07	21.19	52.65	47.47	26.05	27.12	3.87
**S10z**	11.79	35.57	54.73	22.49	8.08	18.79	49.19	43.28	21.60	18.75	3.22
**Ssk**	1.39	2.17	4.86	10.40	0.16	7.55	2.37	2.52	4.89	8.35	−0.11
**Sdq**	0.21	1.30	1.44	0.32	0.20	0.20	1.63	1.36	0.31	0.18	0.10
**FLTt**	23.58	38.37	62.39	26.52	11.07	21.19	52.65	47.47	26.05	27.12	3.87

**Table 5:** Microhardness of samples.

Material	Thickness of Layer, µm	Si_3_N_4_ in Bath g/dm^3^	Graphite in Bath g/dm^3^	HV	±SD
**AW-7075**	-	-	-	**174.75**	4.78
**Ni-P**	10	-	-	**529.4**	5.12
**Ni-P/Si_3_N_4_**	10	0.5	-	**556.25**	20.99
		1	-	**549.75**	23.71
		2	-	**555.75**	15.77
		5	-	**554.5**	11.81
**Ni-P/graphite**	10	-	0.5	**400.25**	53.32
**Ni-P/Si_3_N_4_/graphite**	10	0.5	0.5	**547.25**	1.5
		1	0.5	**534**	7.87
		2	0.5	**672**	32.74
		5	0.5	**598.25**	50.48

**Table 6:** Critical loads for tested Ni-P, Ni-P/Si_3_N_4_ and Ni-P/Si_3_N_4_/graphite coatings.

Material	Si_3_N_4_ in Bath [g/dm^3^]	Graphite in Bath [g/dm^3^]	Critical Load L_c1_ [N]	Critical Load L_c2_ [N]	Coating Removal [mm]
**Ni-P**	-	-	6.05 ÷ 21.27	**13.00 ÷ 33.30**	**5.10 ÷ 5.90**
**Ni-P/Si_3_N_4_**	0.5	-	24.26	**29.62**	**5.00**
	1.0	-	22.01	**37.46**	**5.80**
	2.0	-	22.63	**50.65**	**5.88**
	5.0	-	24.17	**36.96**	**4.11**
**Ni-P/graphite**	-	0.5	2.12	**6.70**	**4.44**
**Ni-P/Si_3_N_4_/graphite**	0.5	0.5	27.36	**34.58**	**8.36**
	1.0	0.5	19.63	**51.57**	**6.69**
	2.0	0.5	37.96	**48.47**	**7.55**
	5.0	0.5	40.25	**56.94**	**8.37**

**Table 7:** Description of the scratch test for Ni-P layer.

Load [N]	Distance “x” [mm]	Description	Type of Failure
<2.01	<0.20	Small longitudinal crack from the beginning.	-
2.01	0.20	Longitudinal cracks extending beyond the outer edges of the crack.	-
4.90	0.50	Single crack with minor chipping.	-
17.23	1.78	The beginning of material uplift at the outer edges of the crack.	
21.27	2.20	Small transverse cracks growing, later exfoliation of the layer in the scratch.	Cohesive
21.56	2.23	Cracks extending from the outer crack edges.	-
33.30	3.45	Larger cracks with chipping.	Adhesive
38.61	4.00	Beginning of increasing layer perforation.	-
46.70	4.84	Cohesive crack with chipping.	Cohesive
49.01	5.08	Removal of the layer.	Adhesive

**Table 8:** Description of the scratch test for Ni-P/graphite (0.5 g/dm^3^) layer.

Load [N]	Distance “x” [mm]	Description	Type of Failure
<2.12	<0.20	From the beginning, small cracks on the tops of sheared agglomerates at the outer edges of the crack.	-
2.12	0.20	A crack with fine chipping of a cohesive type, further on, single rolling of the material.	Cohesive
6.70	0.64	Longitudinal crack, more pronounced, growing, there are cracks with chipping of the layer.	Adhesive
14.90	1.44	In the scratch, a larger longitudinal crack with chipping, flaking and rolling.	Adhesive
22.32	2.16	The beginning of increasing material uplift at the outer edges of the crack, larger single cracks with chipping.	Adhesive
26.85	2.60	Cohesive crack with chipping, increasing perforation of the layer.	Cohesive
45.81	4.44	Destruction of the layer.	Adhesive

**Table 9:** Description of the scratch test for Ni-P/Si_3_N_4_ (0.5 g/dm^3^) layer.

Load [N]	Distance “x” [mm]	Description	Type of Failure
<1.08	<0.10	Minor cracks from scratch.	-
1.08	0.10	Chipping of agglomerates at the outer edges of the crack.	-
3.14	0.30	A single crack with chipping at the bottom of the scratch.	-
5.61	0.54	Flaking and rolling of the material.	-
24.26	2.35	Cracks extending from the outer edges of the crack, increasing material uplift at the outer edges of the crack.	Cohesive
29.62	2.87	Layer chipping and destruction to the substrate, then the layer reappears.	Adhesive
51.57	5.00	Removal of the layer.	Adhesive

**Table 10:** Description of the scratch test for Ni-P/Si_3_N_4_ (1 g/dm^3^) layer.

Load [N]	Distance “x” [mm]	Description	Type of Failure
<6.25	<0.60	Small cracks are visible from the beginning.	-
6.25	0.60	A crack with a small single chip, then small cracks.	-
22.01	2.13	The beginning of cracks extending from the outer edges of the crack.	Cohesive
31.39	3.04	Flaking, rolling, cracks, the beginning of increasing material uplift at the outer edges of the crack.	-
37.46	3.63	Spalling of the layer from the substrate and the first removal of the layer	Adhesive
59.82	5.80	Removal of the layer.	Adhesive

**Table 11:** Description of the scratch test for Ni-P/Si_3_N_4_ (2 g/dm^3^) layer.

Load [N]	Distance “x” [mm]	Description	Type of Failure
<3.75	<0.38	From the beginning of the scratch, a longitudinal crack at the outer edge of the scratch.	-
3.75	0.38	Fine cracks as a result of passing through agglomerates.	-
4.43	0.45	Longitudinal crack in the crack at the inner edge.	-
21.28	2.20	Cracks from the outer edges of the crack, the beginning of material uplift at the edges.	-
22.63	2.34	Beginning of transverse cracks in the scratch.	Cohesive
30.33	3.14	Larger single transverse cracks.	Cohesive
45.93	4.76	Layer perforation.	-
50.65	5.25	Large layer perforation with cracks, chipping and exfoliation.	Adhesive
56.72	5.88	Removal of the layer.	Adhesive

**Table 12:** Description of the scratch test for Ni-P/Si_3_N_4_ (5 g/dm^3^) layer.

Load [N]	Distance “x” [mm]	Description	Type of Failure
<1.92	<0.18	From the beginning, there are longitudinal and transverse cracks resulting from the conduction of the indenter through the agglomerates.	-
1.92	0.18	Small cracks growing in the further part of the scratch.	-
3.95	0.40	Minor chipping in the scratch at the cracks	-
5.39	0.55	Longitudinal cracks in the scratch and at the outer edges.	-
19.84	2.05	Cracks extending from the outer edges of the crack, material uplift at the outer edges.	-
24.17	2.50	Beginning of the transverse fracture network.	Cohesive
33.51	3.47	Larger transverse crack in the scratch.	Cohesive
36.96	3.83	Larger cracks with chipping.	Adhesive
39.67	4.11	Removal of the layer.	Adhesive

**Table 13:** Description of the scratch test for Ni-P/Si_3_N_4_/graphite ((0.5 + 0.5) g/dm^3^) layer.

Load [N]	Distance “x” [mm]	Description	Type of Failure
<4.18	0.40	From the beginning of the scratch, small cracks and detached agglomerates at the outer edges of the scratch.	-
4.18	0.40	Single crack with chipping and rolling.	-
27.36	2.65	Cohesive-type cracks with chipping in the scratch.	Cohesive
28.08	2.72	Small cracks from the outer edge of the scratch.	-
34.58	3.35	First removal of the layer from the substrate.	Adhesive
86.20	8.36	Complete removal of the layer.	Adhesive

**Table 14:** Description of the scratch test for Ni-P/Si_3_N_4_/graphite ((1 + 0.5) g/dm^3^) layer.

Load [N]	Distance “x” [mm]	Description	Type of Failure
<6.27	<0.60	Small cracks are visible from the beginning of the scratch.	-
6.27	0.60	The beginning of increasing material uplift at the outer edges.	-
19.63	1.90	More pronounced transverse cracks with fine chipping	Cohesive
33.85	3.28	Cracks, peeling and rolling.	-
40.24	3.90	Larger single cracks with cohesive spalling, flaking and rolling.	Cohesive
51.57	5.00	First layer removal.	Adhesive
68.99	6.69	Complete removal of the layer.	Adhesive

**Table 15:** Description of the scratch test for Ni-P/Si_3_N_4_/graphite ((2 + 0.5) g/dm^3^) layer.

Load [N]	Distance “x” [mm]	Description	Type of Failure
<8.90	<0.86	Small cracks in the scratch and along the outer edges of the scratch are visible from the beginning of the scratch.	-
8.90	0.86	Larger longitudinal cracks on the border of the crack edge.	-
33.94	3.29	Minor cracks from the outer edges of the scratch.	-
37.96	3.68	Large crack with cohesive chipping.	Cohesive
48.47	4.70	Layer chipping off the substrate.	Adhesive
53.62	5.20	Cracks and minor chipping.	Adhesive
77.84	7.55	Removal of the layer.	Adhesive

**Table 16:** Description of the scratch test for Ni-P/Si_3_N_4_/graphite ((5 + 0.5) g/dm^3^) layer.

Load [N]	Distance “x” [mm]	Description	Type of Failure
<1.10	<0.10	Small local perforations from the beginning of the scratch.	-
1.10	0.10	Small cracks in the scratch, agglomerates falling off at the outer edges of the scratch.	-
2.85	0.27	Small cracks extending from the outer edges of the crack, a crack with agglomerate chipping.	-
5.22	0.50	Flaking of the material, minor cracks in the scratch.	-
9.24	0.89	Single minor cracks in the scratch.	-
40.25	3.90	Flaking and rolling, a greater number of cracks extending from the outer edges of the crack.	Cohesive
56.94	5.52	The beginning of single larger cracks with spalling and flaking.	Adhesive
72.18	7.00	Beginning of increasing layer perforation.	-
86.30	8.37	Removal of the layer.	Adhesive

## Data Availability

Not applicable.
